# Gastrointestinal Histoplasmosis as an Obstructing Ileocecal Mass

**DOI:** 10.7759/cureus.12277

**Published:** 2020-12-25

**Authors:** Zaid Nawaz, Shabiah Martin, Ari R Reichstein

**Affiliations:** 1 Department of General Surgery, Edward Via College of Osteopathic Medicine, Blacksburg, USA; 2 Colorectal Surgery, Allegheny Health Network, Pittsburgh, USA

**Keywords:** gastrointestinal histoplasmosis, ileocecal mass, disseminated histoplasmosis, small bowel obstruction, tumor necrosis factor inhibitor medications, biologic agents

## Abstract

Histoplasmosis is a self-limiting and asymptomatic disease in immunocompetent individuals. Patients in an immunocompromised state are susceptible to disseminated disease. We present a case of a 60-year-old male with a history of psoriatic and rheumatoid arthritis treated with a tumor necrosis factor inhibitor (adalimumab), who presented with abdominal pain and was found to have gastrointestinal histoplasmosis as an obstructing ileocecal mass. Although gastrointestinal involvement is common in disseminating disease, symptomatic involvement is rare. This case presentation has implications in rheumatological patients on biologic medications.

## Introduction

Histoplasmosis is a fungal infection endemic to the Ohio River valley [1]. It can present clinically in many ways, including asymptomatic, acute and chronic pulmonary infections, or disseminated disease. Infection occurs almost exclusively by inhalation of the airborne conidia via bird or bat droppings [2]. In the majority of cases, individuals are asymptomatic and are able to clear the disease [3]. The progression to disseminated disease indicates a non-functional cell-mediated immune response. Patients that are faced with the disseminated disease include those that are in an immunocompromised state, including patients with HIV/AIDS, recipients of bone marrow or organ transplantation, those on tumor necrosis factor inhibitors, those taking corticosteroids, patients with genetic diseases, and patients with hematologic cancers [4]. Histoplasma can be found anywhere in the gastrointestinal tract, from the mouth to the anus. The most commonly involved sites are the colon and the terminal ileum [5]. Here we present a case of an elderly male with disseminated histoplasmosis with prominent manifestations of the small bowel and colonic involvement.

## Case presentation

**Figure 1:**
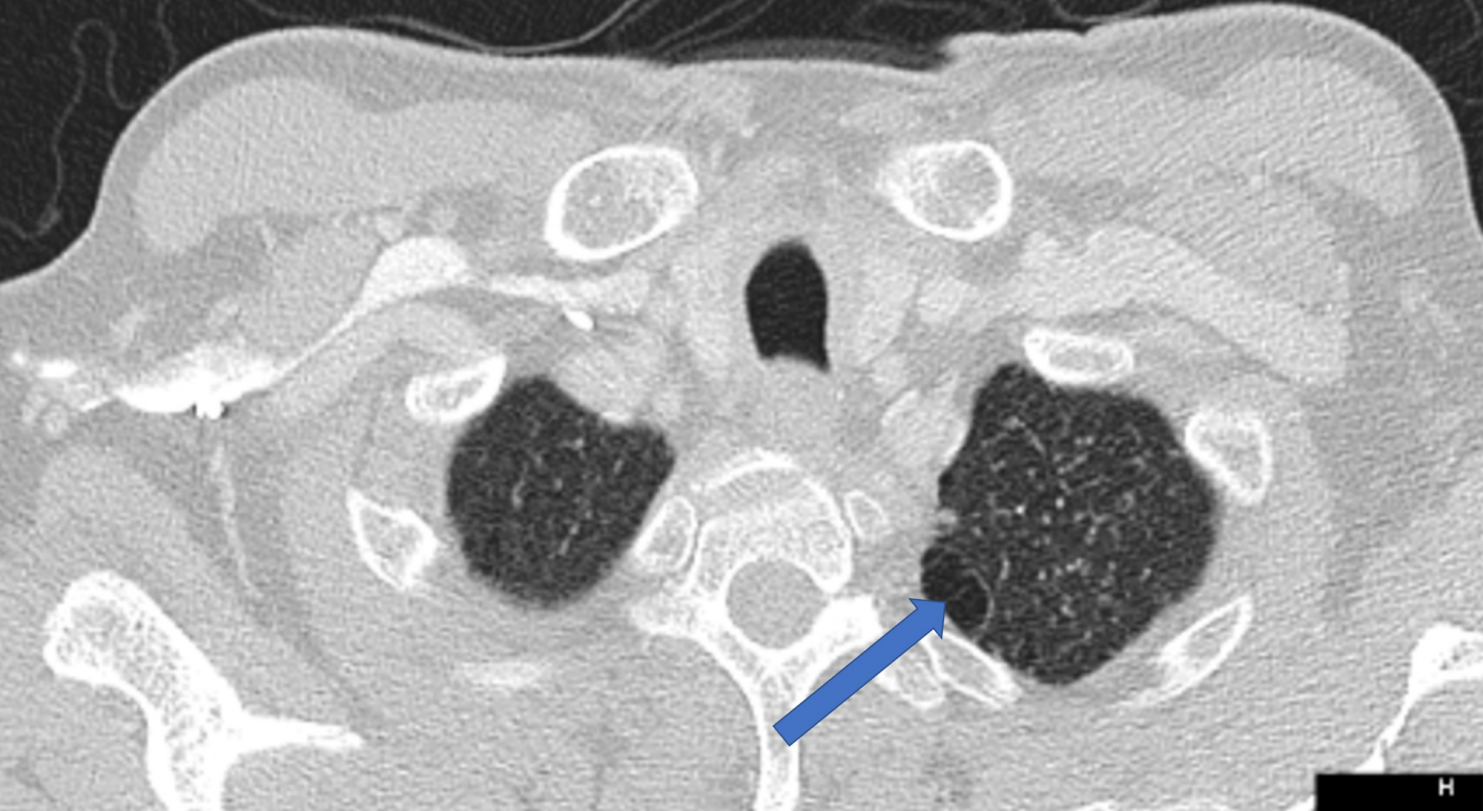
CT scan of chest on initial admission Pulmonary nodule seen in the left lobe of the lung (arrow)

**Figure 2:**
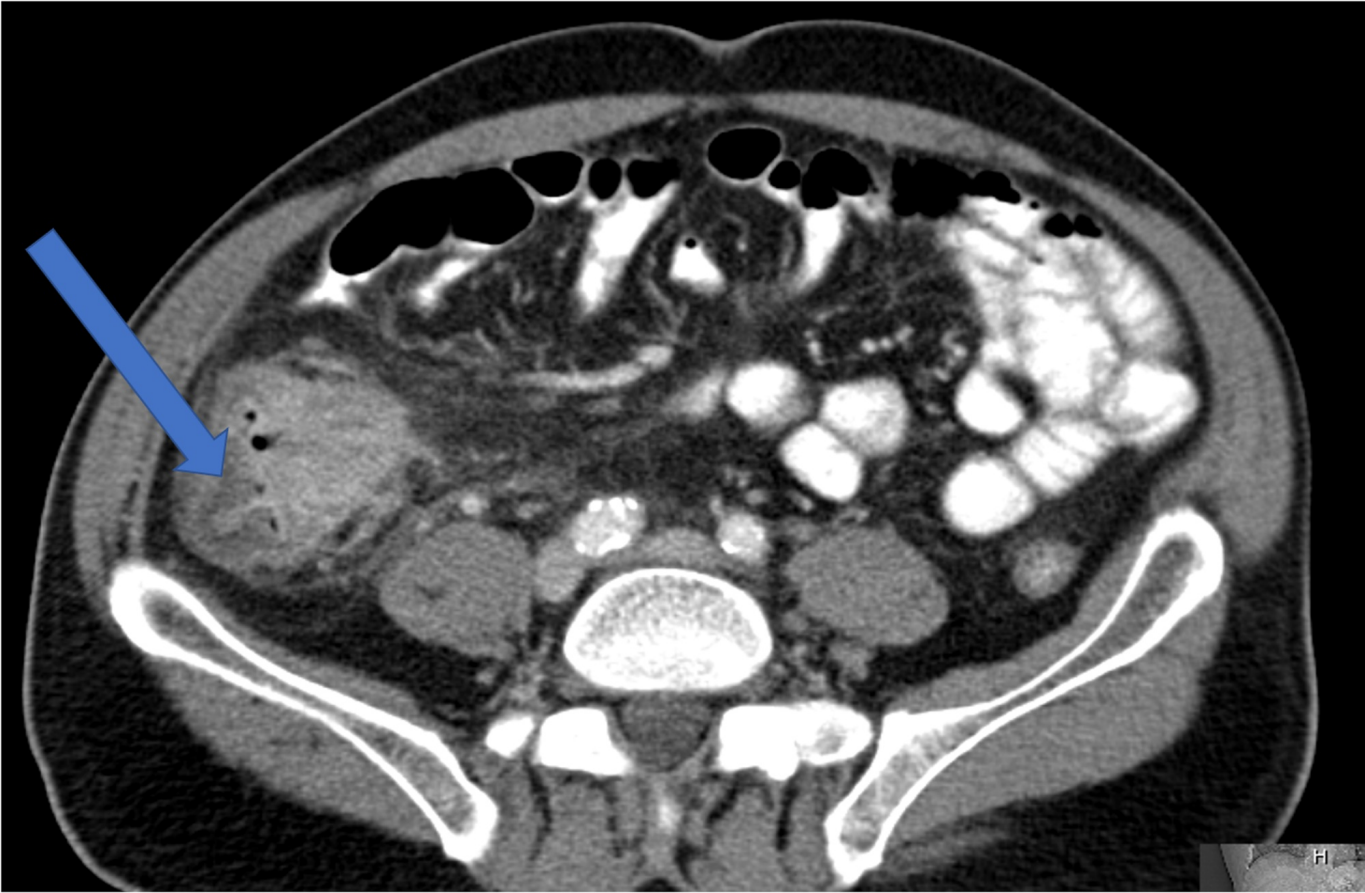
CT abdomen/pelvis scan on initial admission Mass (arrow) causing obstruction, possibly in the cecum or distal ileum

A 60-year-old male with a past medical history significant for psoriatic and rheumatoid arthritis for which he was receiving adalimumab and methotrexate presented with symptoms of bowel obstruction, including abdominal pain, nausea, and vomiting. On further workup, blood cultures were significant for disseminated histoplasmosis, and CT scan of chest, abdomen, and pelvis demonstrated pulmonary nodules (Figure 1) and an ileocecal mass (Figure 2), pathology of which was consistent for histoplasmosis. The patient was initially managed conservatively for his bowel obstructions and was started on intravenous amphotericin b. When his cerebrospinal fluid (CSF) was negative for histoplasmosis, he transitioned to oral itraconazole. The patient was subsequently discharged home on hospital day 17 after his obstruction had resolved.

**Figure 3:**
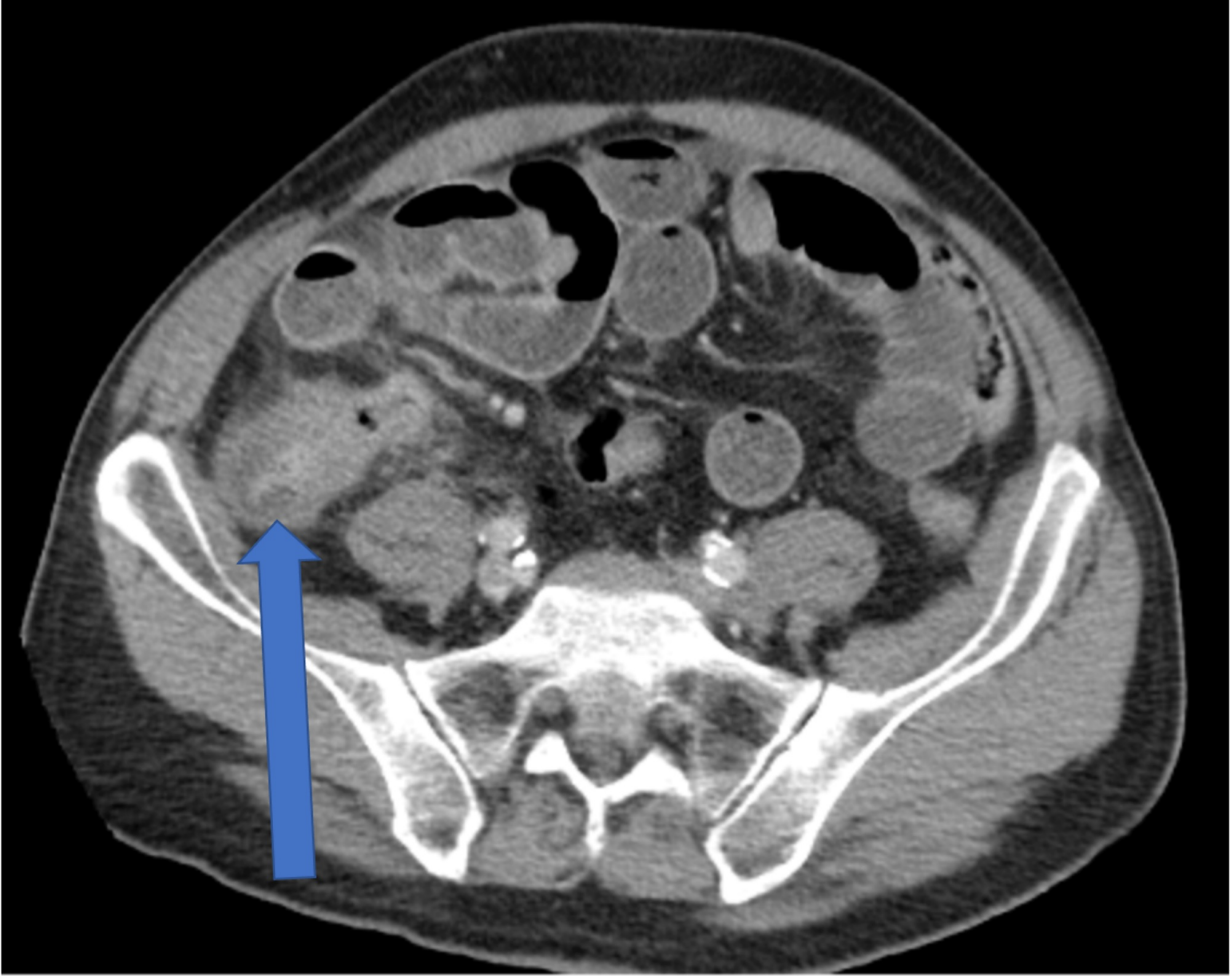
CT abdomen/pelvis on the second admission Ileocecal mass (arrow) causing obstruction

**Figure 4:**
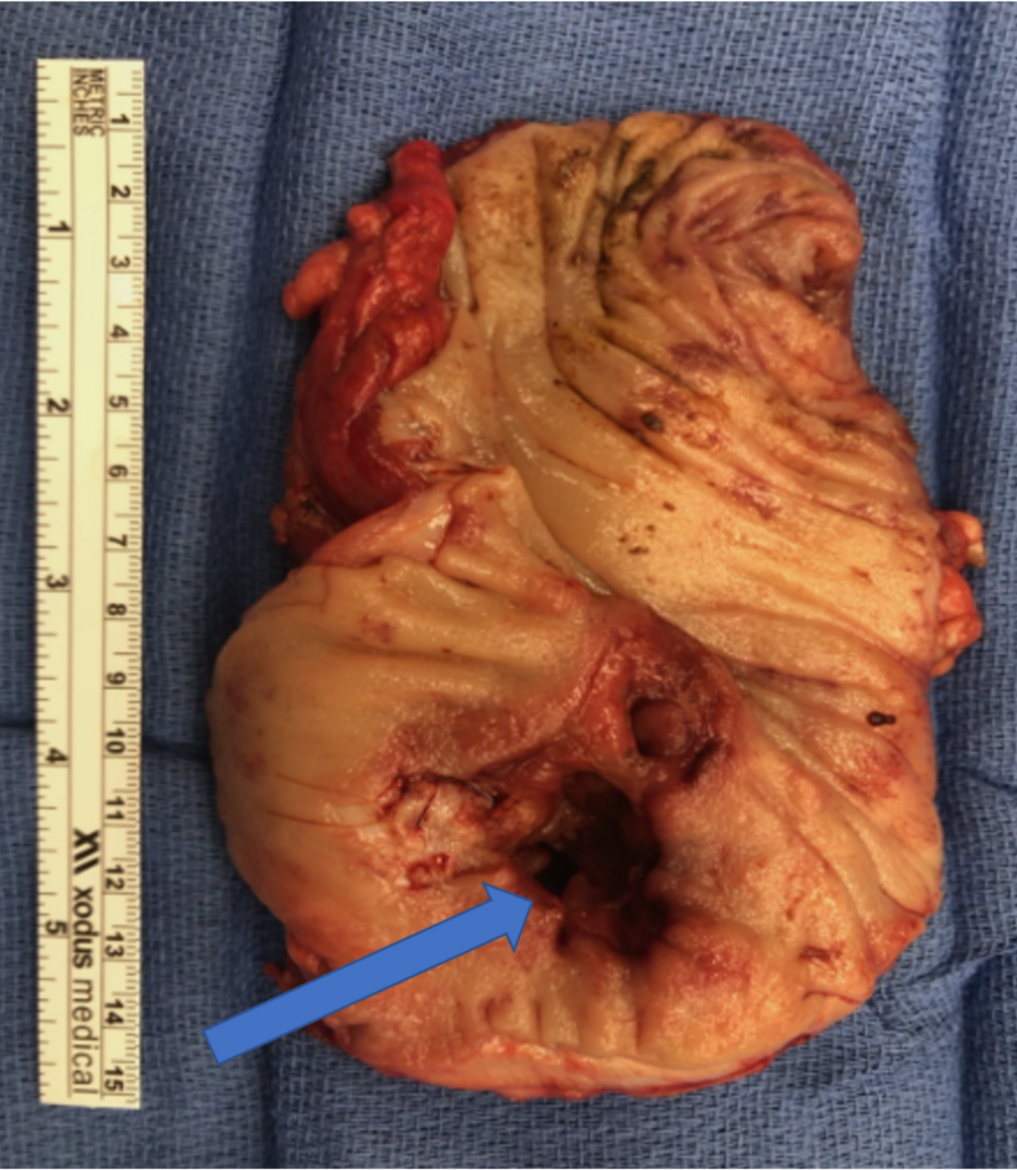
Ileocecal mass (arrow) surgically excised

Less than two weeks later, the patient presented to the emergency room complaining of abdominal pain, nausea, vomiting, and obstipation. An abdominal CT scan (Figure 3) was obtained and showed high-grade small bowel obstruction at the terminal ileum and a possible cecal mass. He was taken to the operating room for surgery to remove the ileocecal mass (Figure 4) and had a laparoscopic assisted right colectomy with end ileostomy. Pathological analysis showed necrotizing granulomatous inflammation and yeast forms consistent with Histoplasma. The postoperative course was uneventful. The patient was started on intravenous amphotericin b and transitioned to a two-week course of itraconazole. He progressed as expected and was safely discharged home.

## Discussion

Histoplasma capsulatum is a dimorphic fungus endemic to Ohio, Mississippi, Missouri river valleys in the United States and some river valleys in Central America [1]. The spores are inhaled into the lungs, where it transforms from a mold state into a yeast. Alveolar macrophages phagocytize these yeasts and start a cascade of events that helps to corral the pathogen. Antigen-presenting cells and CD-4 T helper cells secrete cytokines, including IL-12 and interferon-gamma, to activate macrophages [6]. In turn, they then secrete tumor necrosis factor (TNF) inhibitor to induce the formation of granulomas and wall-off pathogens from spreading. Depletion of these factors prevents the development of effective cell-mediated immunity against an organism like Histoplasma, leading to disseminated disease.

Risk factors for disseminated disease are primarily for those individuals who are immunocompromised. These include patients that have HIV/ AIDS as their cell-mediated immunity wanes over time [4]. Individuals on immunosuppressive medications, including corticosteroids, interferon-gamma inhibitors, disease-modifying anti-rheumatic drugs (DMARDS), anti-TNF inhibitors including other biologics, etc., are all at a greater risk for disseminated Histoplasma. It has long been appreciated that these medications confer an increased risk for tuberculosis, but studies have shown that the risk is also increased to disseminated fungal infections [7]. Our patient was on adalimumab and methotrexate for psoriatic/rheumatoid arthritis. Anti-TNF medications are prevalent in groups, including those affected by rheumatoid arthritis, inflammatory bowel disease, psoriasis, ankylosing spondylitis, vasculitis, and uveitis. Studies have shown that although it is rare for individuals to get disseminated histoplasmosis from these medications, it has happened, and the likelihood of disseminated disease is greater in those on biologics (anti-TNF, interferon-gamma inhibitors, etc.) [8]. Individuals on disease-modifying antirheumatic drugs are also at an increased risk for disseminated histoplasmosis, as these medications also cause defective cellular immunity [9].

Disseminated histoplasmosis is a rare disease that presents almost exclusively in the immunocompromised state [5]. It can affect multiple organ systems, including the skin, central nervous system, lungs, liver, spleen, adrenal glands, and gastrointestinal tract through to the colon [10]. Gastrointestinal involvement occurs in 70% to 90% of cases of disseminated disease, although in the vast majority of cases, the patients are asymptomatic. It manifests symptomatically in 3%-12% of patients who develop disseminated histoplasmosis [5]. Pathologic findings can include mucosal ulcerations, gastrointestinal bleeding, submucosal nodules, diffuse lymphohistiocytic infiltration of the bowel wall, diarrhea, obstructing mass, and bowel perforation [11]. Our patient had a fungating mass with necrotizing granulomatous inflammation, causing obstructive symptoms in the terminal ileum and proximal cecum. The terminal ileum and colon are the most frequently involved organs [11]. Others can present with severe inflammation and bloody diarrhea mimicking inflammatory bowel disease. The probable reason that our patient presented with obstructing symptoms and not diarrhea is due to the localized granulomatous inflammation resulting in fungating mass.

Disseminated histoplasmosis is fatal if left untreated [6]. Treatment includes amphotericin b and itraconazole, which are highly effective. Our patient had an initial antibiotic course of both medications, which resolved his symptoms and cleared his disease as his Histoplasma antigen levels showed that he was negative for the disease. Surprisingly, the patient returned shortly after with such a fungating mass that caused obstructive symptoms. Improvements in the disease are usually seen within a week in most patients, with relapse occurring in patients who are persistently immunosuppressed [6]. Our patient was off of his adalimumab and methotrexate for more than a month. Adalimumab has a half-life of approximately two weeks, with methotrexate being only a couple of hours [12,13]. Further treatments have not been determined in the literature on recurrent small bowel obstruction caused by disseminated histoplasmosis. Typically patients undergo bowel rest for initial small bowel obstruction, with surgery as the option for complete obstruction, especially for those with strictures [14]. Since our patient had initially gone through medical management and presented with worsening symptoms so soon, we decided to pursue surgical management alongside a combination of amphotericin b and itraconazole.

## Conclusions

Disseminated histoplasmosis is a rare disease typically seen in individuals with HIV/AIDS. It is infrequently seen in patients on anti-TNF medications. Gastrointestinal histoplasmosis is symptomatic only in a small percentage of individuals who have disseminated disease. In this case presentation, our patient had recurrent disseminated disease marked with symptomatic gastrointestinal histoplasmosis as a fungating mass, causing obstruction requiring surgical management. In conclusion, clinicians should be aware of the potential side effect of biologic medications and their implications on disseminated histoplasmosis. Initial clearance of histoplasmosis does not always mean that the patient is free from recurrence of the disease, which can manifest in a more severe form. It is also important to decide further treatment options for recurrent disease as histoplasmosis can cause severe morbidity and mortality.
